# The “Oblique Popliteal Ligament”: A Macro- and Microanalysis to Determine If It Is a Ligament or a Tendon

**DOI:** 10.1155/2012/151342

**Published:** 2012-05-21

**Authors:** Brion Benninger, Taylor Delamarter

**Affiliations:** ^1^Department of Medical Anatomical Sciences, Western University of Health Sciences, COMP-Northwest, 200 Mullins Way, Lebanon, OR 97355, USA; ^2^Department of Family Practice, Western University of Health Sciences, COMP-Northwest, 200 Mullins Way, Lebanon, OR 97355, USA; ^3^Samaritan Health Services Orthopaedics, Residency Faculty, Corvallis, OR 97330, USA; ^4^Samaritan Health Services General Surgery, Residency Faculty, Corvallis, OR 97330, USA; ^5^Department of Oral Maxillofacial Surgery, Oregon Health & Science University, 611 SW Campus Drive, Portland, OR 97239, USA; ^6^Department of Surgery, Oregon Health & Science University, 611 SW Campus Drive, Portland, OR 97239, USA; ^7^Department of Orthopaedics & Rehabilitation, Oregon Health & Science University, 611 SW Campus Drive, Portland, OR 97239, USA

## Abstract

*Introduction*. This study investigated the importance of the “oblique popliteal ligament” (OPL), and challenges its alleged ligament status. The currently named OPL is indigenous to the distal semimembranosus (SMT); therefore, by definition is not a ligament inserting from bone to bone. Clinically, a muscle-tendon unit is different then a ligament regarding proprioception and surgery. *Methods*. Literature search was conducted on texts, journals and websites regarding the formation of the OPL. Dissection of 70 knees included macro analysis, harvesting OPL, distal SMT and LCL samples and performing immunohistochemistry to 16 knees with antibody staining to the OPL, distal SMT and LCL. *Results*. All but one text claimed the OPL receives fibers from SMT. Macro dissection of 70 knees revealed the OPL forming from the distal SMT (100%). Microanalysis of OPL, distal SMT and LCL samples from 16 knees demonstrated expression of nervous tissue within selected samples. *Discussion*. No journals or texts have hypothesized that the OPL is a tendon. Clinically it is important we know the type of tissue for purposes of maximizing rehabilitation and surgical techniques. *Conclusion*. This study suggests the OPL be considered the oblique popliteal tendon as a result of the macro and micro evidence revealed.

## 1. Introduction

The posterior aspect of the knee has been increasingly studied because of its clinical relevance. Surgeons, biomechanists, physical therapists, all health care providers dealing with the musculoskeletal system, and anatomists need to have a definitive and precise understanding of the structures of the posteromedial knee. A previous study conducted by the authors identified the clinical importance, morphology, and accurate terminology of the distal semimembranosus muscle tendon unit (SMTU) [1]. This study also revealed that the currently named oblique popliteal ligament (OPL) was indigenous to the SMTU and, therefore, by definition is not a ligament inserting from bone to bone. This is clinically important because of the proprioception of a tendon versus a ligament, which may suggest a greater role by the distal semimembranosus tendon in posterior knee stability.

With regard to the literature regarding the oblique popliteal ligament, Woodburne's *Essentials of Human Anatomy* states that it is formed from the fibers of the distal semimembranosus tendon [2]. All other anatomical texts and atlases that consider or depict the OPL state that the distal semimembranosus tendon contributes fibers to the OPL [2–20]. Though the majority of the texts and journal papers describe the SMTU contributing to the OPL [21–32], none have hypothesized that this ligament is indigenous to the SMTU, therefore, a tendon by the true sense of the definition.

In order to provide further evidence towards this hypothesis, histological studies of the SMTU, OPL, and a well-defined ligament of the knee were needed. There have been previous histological studies completed on the various structures of the knee, the majority of which have primarily focused on the specific type of nerve ending present within these deep structures, particularly the cruciate ligaments and the menisci [33–41]. None have specifically looked at the histology of the OPL, and immunohistochemistry staining specific to the neuronal axons has not been conducted on any deep structures of the knee. Therefore, the authors conducted immunohistochemistry staining with antibodies specific to neuronal axons on the SMT, OPL, and the lateral collateral ligament (LCL) of the knee. The staining conducted allowed the author to compare the histology and neuronal components of the SMTU with the OPL and a well-defined ligament of the knee, such as the LCL. The objective of this study was to conduct a macro- and microanalysis investigation of the OPL and challenge its alleged ligament status.

## 2. Materials and Methods

A literature search was conducted on anatomical and specialty texts, atlases, journals, and websites regarding the morphology of the distal semimembranosus muscle tendon unit and oblique popliteal ligament. Deep dissections were performed on 43 embalmed human cadavers (23 M and 20 F, age: 55–89, average: 79.6 yrs), 70 knees in total (39 Rt and 41 Lt), to reveal the SMTU and its final attachments. Exclusion criteria are amputation, knee replacement, or any gross damage to the knee joint. The most distal portion of the SMTU was reflected medial to lateral in order to analyze whether or not the alleged oblique popliteal ligament is a continuation of the distal SMTU, or if it was a structure attaching from bone to bone. The OPL's distal (medial) and proximal (lateral) attachments were analyzed. Immunohistochemistry staining was performed on the SMTU, OPL, and LCL using the following protocols: PGP9.5 staining of human tendon/ligament sections with rabbit anti-PGP9.5 (Accurate Chemical)/goat anti-rabbit biotinylated (Vector), neuronal class III *β*-tubulin (NCT), and staining of human tendon/ligament sections with rabbit anti-NCT (Covance)/goat anti-rabbit biotinylated (Vector).

## 3. Results

**Figure 1:**
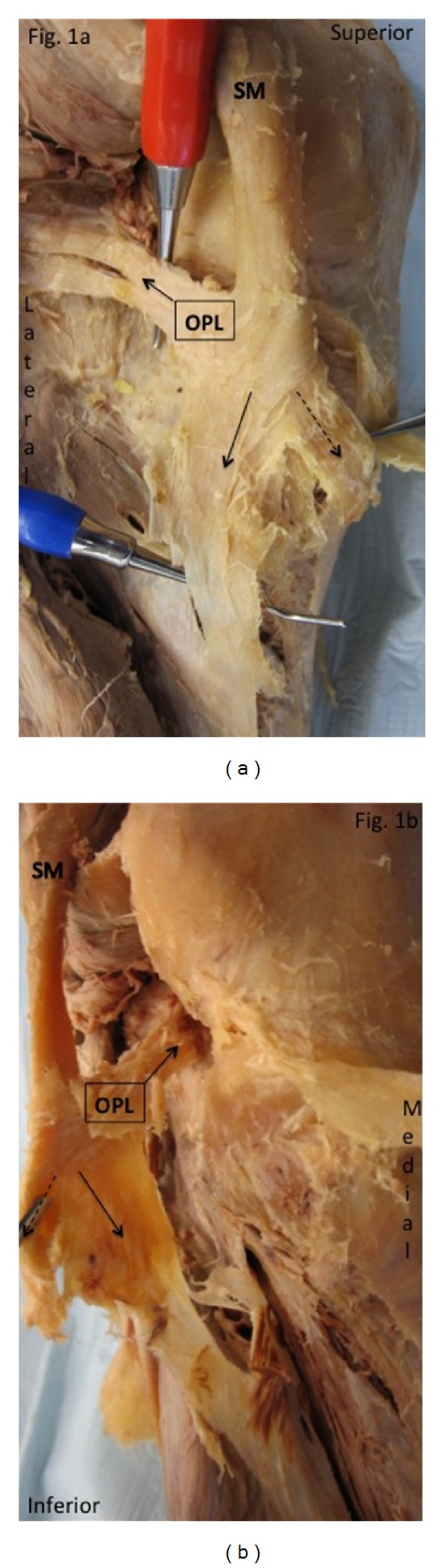
(a) Deep dissection of the left posteromedial knee revealing the distal semimembranosus muscle tendon unit (SMTU) and oblique popliteal ligament (OP). SM: semimembranosus muscle. (b) Left SMTU reflected revealing that the alleged OPL is indigenous to the SMTU. Arrow: direct arm of the SMTU; dashed arrow: anterior arm of the SMTU.

**Figure 2:**
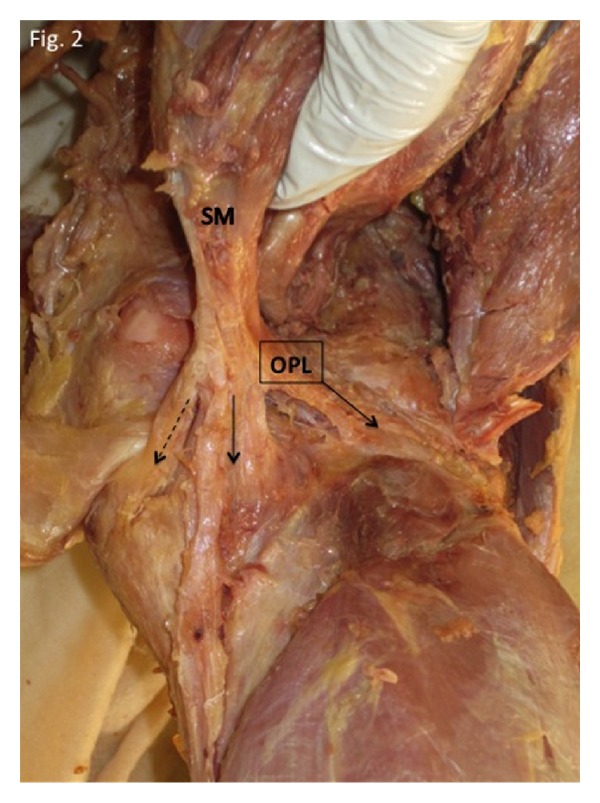
Deep dissection of the right posterior knee revealing the oblique popliteal ligament (OPL). SM: semimembranosus muscle. Arrow: direct arm of the SMTU; dashed arrow: anterior arm of the SMTU.

**Figure 3:**
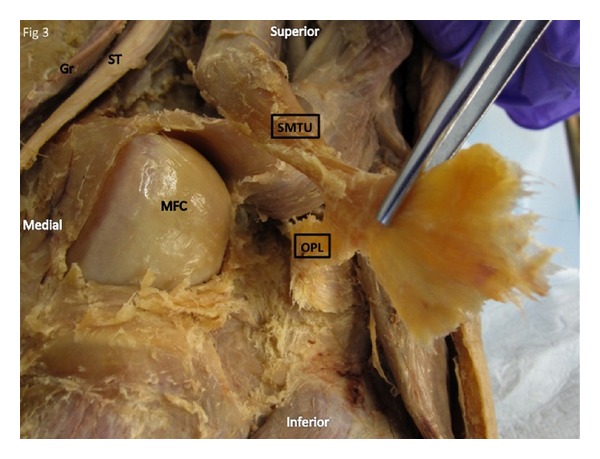
Deep dissection of the right posteromedial knee. Distal semimembranosus muscle tendon unit (SMTU) reflected revealing that the alleged oblique popliteal ligament is indigenous to the distal semimembranosus tendon. SM: semimembranosus muscle, SMTU: distal semimembranosus muscle tendon unit, OPL: oblique popliteal ligament, MFC: medial femoral condyle, ST: semitendinous muscle, Gr: gracilis muscle.

**Figure 4:**
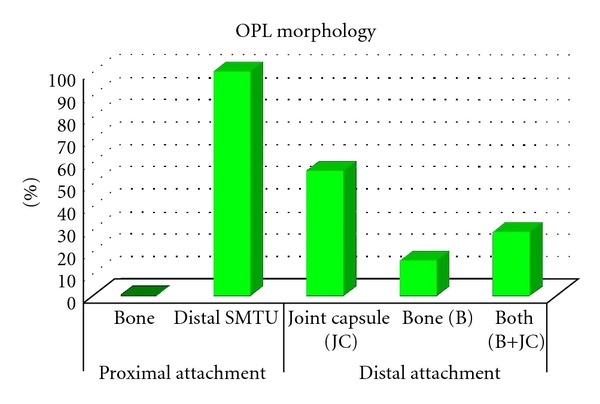
Oblique popliteal ligament morphology (OPL). Results: proximal and distal attachments.

**Figure 5:**
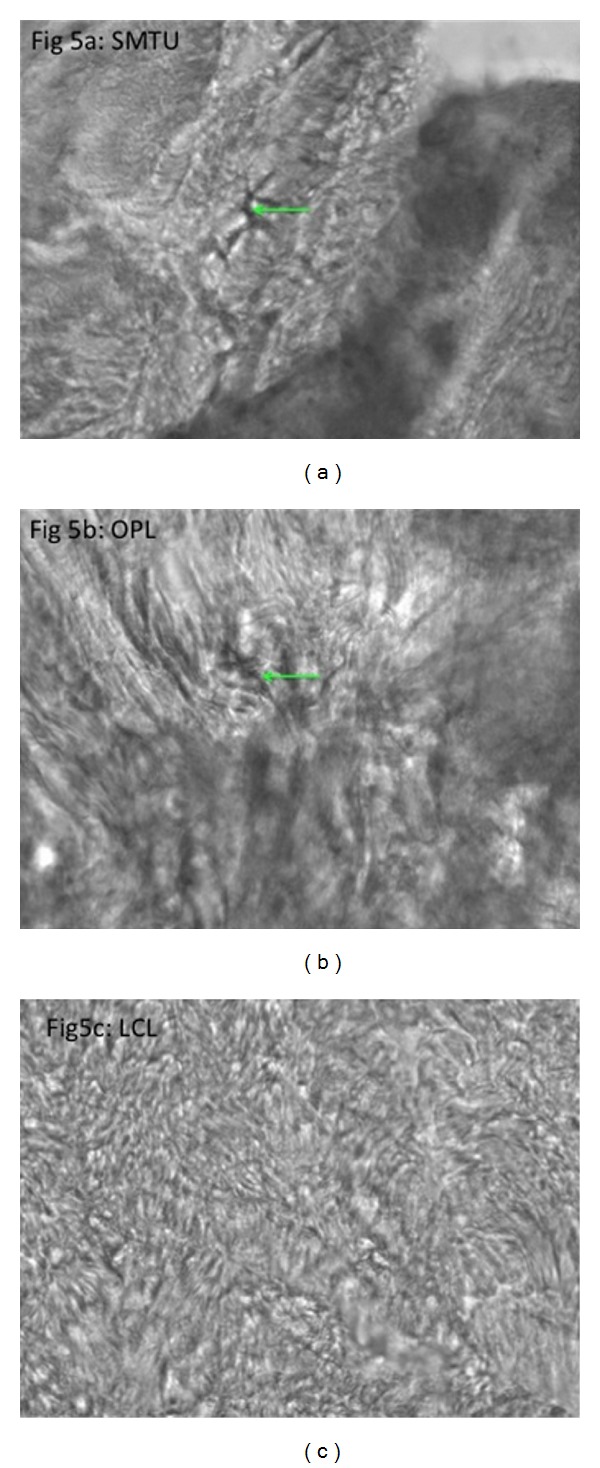
Light microscope view (20x) of PGP9.5 stain revealing neuronal axon (arrow). (a) Distal semimembranosus muscle tendon unit (SMTU). (b) Oblique popliteal ligament (OPL). (c) Lateral collateral ligament of the knee (LCL).

**Figure 6:**
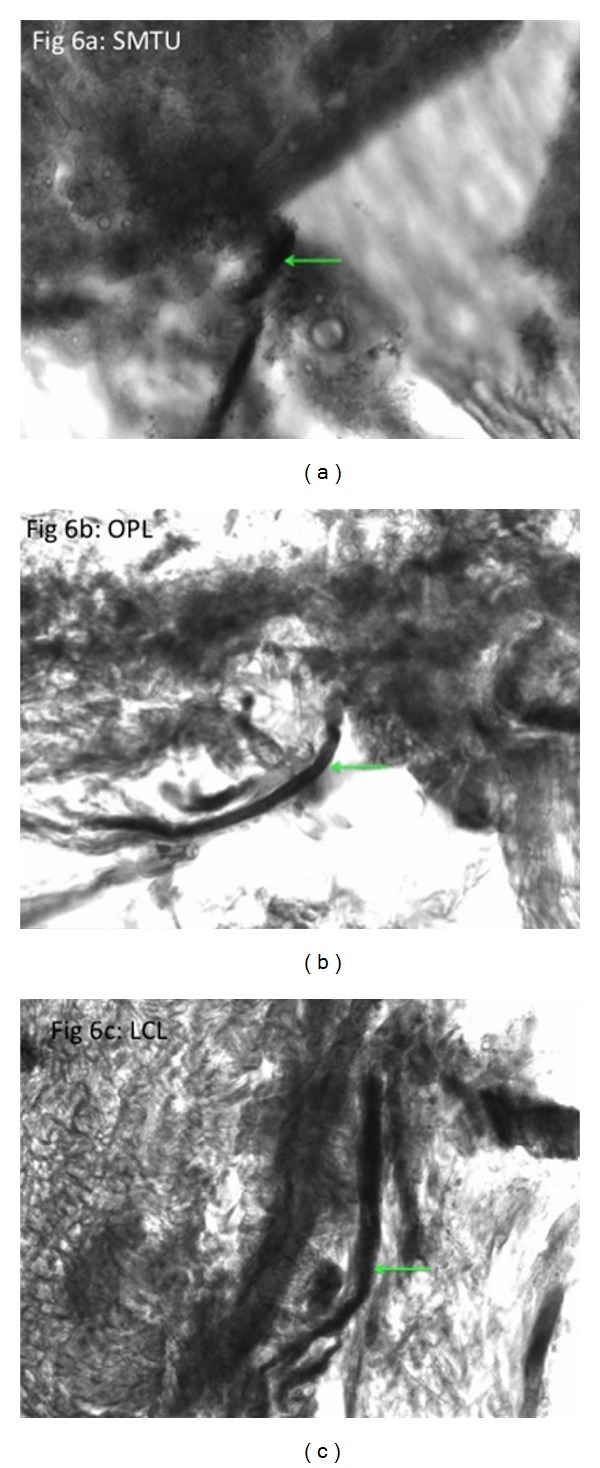
Light microscope view (20x) of *β*-tubulin stain revealing neuronal axon (arrow). (a) Distal semimembranosus muscle tendon unit (SMTU). (b) Oblique popliteal ligament (OPL). (c) Lateral collateral ligament of the knee (LCL).

**Figure 7:**
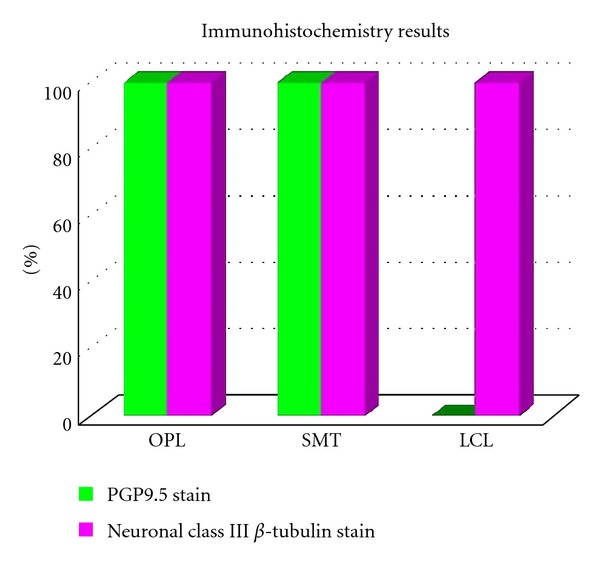
Immunohistochemistry results of PGP9.5 staining of human tendon/ligament sections with rabbit anti-PGP9.5 (Accurate Chemical)/goat anti-rabbit biotinylated (Vector) and neuronal class III *β*-tubulin (NCT), staining of human tendon/ligament sections with rabbit anti-NCT (Covance)/goat anti-rabbit biotinylated (Vector).

Literature search revealed that 11 of the 19 anatomical texts and atlases that consider or depict the OPL state that the distal semimembranosus tendon contributes fibers to the OPL [2–20]. A much higher percentage was found in orthopedic or radiologic specialty articles (11 of 12 stated that the distal semimembranosus tendon contributes fibers to the OPL) [21–32] (see Table 1). Deep dissections revealed that the alleged oblique popliteal ligament's distal (medial) attachment originated from the SMTU in 100% of 70 knees. Its proximal (lateral) attachment was inserted into the joint capsule in 39/70, bone in 11/70, and both joint capsule and bone in 20/70 knees (see Figures 1(a), 1(b), 2, 3, and 4). Immunohistochemistry staining using rabbit anti-PGP9.5/goat anti-rabbit biotinylated revealed a positive stain for neuronal axons in both the SMT and the OPL and a negative stain in the LCL. Immunohistochemistry staining using neuronal class III *β*-tubulin (NCT) staining of human tendon/ligament sections with rabbit anti-NCT/goat anti-rabbit biotinylated revealed a positive stain for neuronal axons in each of three tissue types, OPL, SMT, and LCL (see Figures 5(a), 5(b), 5(c), 6(a), 6(b), 6(c) and 7). 

## 4. Discussion

Despite the fact that nearly 60 percent of anatomical texts and atlases as well as over 90 percent of specialty journal articles state that the distal semimembranosus tendon contributes fibers to the oblique popliteal ligament; none have hypothesized that this structure is itself a tendon [1–31]. A macroanalysis using deep dissection of the posterior knee revealed that the OPL's distal (medial) attachment originated from the SMTU in 100% of the knees. This provided evidence in support of the author's hypothesis; however, a microanalysis was also necessary to propitiate these findings. This study was the first to conduct a histological microanalysis of the OPL. 

There have been previous studies that have used various staining protocols on the deep tissue of the knee, namely, the cruciate ligaments, menisci, and the medial collateral ligament [33–40]. The majority of this research conducted histological studies specifically targeting the morphology of nerve endings in these tissues. This was the first known study to use immunohistochemistry staining with an antibody specific to neuronal axons in the deep tissue of the knee. This was also the first study to utilize any staining protocol on the OPL.

The microanalysis of the tendon properties using rabbit anti-PGP9.5/goat anti-rabbit biotinylated immunohistochemistry staining revealed neuronal axons in both the SMTU and the OPL and displayed similar histological patterns in both structures [33]. The LCL did not display a positive result for this stain and had a markedly different histology to both the OPL and SMT. Furthermore, the positive stain for neuronal axons provides grounds that Golgi tendon organs, nervous tissue specific to tendons, may be located in the OPL. These facts confirm the author's hypothesis that this structure is a tendon.

The authors are not aware of a stain specific to Golgi tendon organs. However, in pursuit of providing increased evidential proof for a change in terminology, the authors conducted a different, more definitive immunohistochemistry stain for neuronal axons using neuronal class III *β*-tubulin (NCT) with rabbit anti-NCT/goat anti-rabbit biotinylated. Though the histology of both the SMTU and the OPL was once again quite similar and vastly different from that of the LCL, the stain revealed a positive stain for neural tissue in all three structures: the OPL, SMTU, and LCL. This result does not nullify the results obtained from the PGP9.5 stains; however, it forced the authors to question whether or not immunohistochemistry staining for neural tissue within these structures is the most viable method for differentiating tendon from ligament.

The macroanalysis of the distal SMTU provides undeniable evidence that the OPL is indigenous to this tendon. The immunohistochemistry used in this study is proven to provide definitive results for neuronal axons within tissue samples [42] and was the first to demonstrate that there is nervous tissue within the OPL. Despite the inconclusive results of the final immunohistochemistry stains, the macro- and microevidence that the oblique popliteal ligament is not a ligament at all is overwhelming. This evidence has led the authors to propose a nomenclature change for this structure, naming it the oblique popliteal tendon.

## 5. Conclusion

The macroanalysis of the OPL revealed unequivocally it is indigenous to the distal SMTU. The microanalysis using an immunohistochemistry stain with PGP9.5 revealed a positive result for neuronal axons within both the SMT and OPL. Further microanalysis using an immunohistochemistry stain with *β*-tubulin revealed a positive stain for neuronal axons in the SMT, OPL, and LCL. Though the latter result leads the authors to question the validity of differentiating tendon from ligament using this particular immunohistochemistry stain, the macroanalysis results are overwhelming, and the microanalysis reveals striking similarities in the histology of both the OPL and SMT. The authors strongly suggest that the oblique popliteal ligament be renamed the oblique popliteal tendon (O) due this macro- and microanalysis study. Clinically, this study improves terminology accuracy and medical international language, allowing for better understanding of successful rehabilitation methods and rationale for current and future surgical procedures.

## Figures and Tables

**Table 1:** Contribution to the OPL from the distal semimembranosus tendon via anatomical texts and atlases and specialty journals.

Anatomical Texts and Atlases	Semimembranosus contributes fibers to the oblique popliteal ligament (OPL)	Speciality journals	Semimembranosus contributes fibers to the oblique popliteal ligament (OPL)
Anatomy as a Basis for Clinical Medicine [11]	X	Some Aspects of Functional Anatomy of The Knee Joint [12]	

Atlas of Human Anatomy [13]		The Supporting Structures and Layers on The Medial Side of The Knee [31]	X (OPL)

Anatomy for Surgeons [43]	X	Anatomy of The Medial Part of The Knee [26]	

BRS Gross Anatomy [5]	X	Anatomy of The Posterior Aspect of The Knee [27]	X (OPL)

Clemente Anatomy [6]		Distal Semimembranosus Complex: The Normal MR Anatomy, Variants, Biomechanics and Pathology [21]	X (OPL)

Clinical Anatomy [9]	X	Tendinous Insertion of Semimembranosus Into The Lateral Meniscus [24]	X (OPL)

Clinical Anatomy by Systems [18] Atlas	X	Posteromedial Corner of The Knee: MR Imaging with Gross Anatomic Correlation [28]	X (OPL)

Clinical Orthopaedic Rehabilitation [4]		Avulsion of The Posteromedial Tibial Plateau by The Semimembranosus Tendon: Diagnosis with MR Imaging [32]	X (contribution to OPL)

Color Atlas and Textbook of Human Anatomy [10]	X	The Posteromedial Corner of The Knee: Medial Injury Patterns Revisited [30]	X (OPL)

Essential Clinical Anatomy [15]	X	Hamstring Muscle Complex: An Imaging Review [25]	X (OPL/arcuate)

Essentials of Human Anatomy [2]	X	A Note on The Semimembranosus Muscle [22]	X (OPL)

Grant's Atlas of Anatomy [3]	X	Semimembranosus Tendon Viewed through an Isolated Medial Meniscus Capsular Avulsion: A Case Report [29]	X (OPL/ligament of Winslow)

Gray's Anatomy 40th ed	X		

Gray's Anatomy for Students [8]			

Gray's Atlas of anatomy [7]			

Gross Anatomy in the practice of medicine [17]			

Lippincott Williams & Wilkins Atlas of Anatomy [20]			

Sports Injury Assessment and Rehabilitation [16]			

Surgical Atlas of Sports Medicine [14]	X		
